# CULTURE AND COGNITION: RISK FACTORS FOR ALZHEIMER’S DISEASE IN US HONOR CULTURES

**DOI:** 10.1093/geroni/igae098.2584

**Published:** 2024-12-31

**Authors:** Erin Harrington, Jarrod Bock

**Affiliations:** University of Wyoming, Laramie, Wyoming, United States; University of Wyoming, Laramie, Wyoming, United States

## Abstract

Alzheimer’s disease is the sixth leading cause of death among all Americans. As the prevalence of Alzheimer’s disease and related dementias (ADRD) continues to grow, researchers and clinicians are ever cognizant of cultural predictors that may contribute to ADRD risk. One cultural approach not yet examined within the context of ADRD is honor cultures (i.e., primarily southern and western states), where reputation management and defense are paramount. Normative behaviors within honor cultures (e.g., risk taking, military involvement, intimate partner violence) may heighten ADRD risk, particularly related to traumatic brain injury (TBI) and subjective cognitive decline (SCD). Further, the lack of general and mental health resources and stigma toward help-seeking in honor cultures likely leave these outcomes untreated. As such, the present study used statewide data collected between 2009-2019 to examine links between statewide honor-orientation and unintentional TBI death rates, frequency of SCD, and AD death rates. Honor-orientation was a significant predictor of unintentional TBI death rates (ß =.44, p <.001), SCD frequency (ß =.58, p =.004), and AD death rates (ß =.41, p =.048), over-and-above state-level covariates (i.e., rurality, temperature, economic conditions, healthcare access). Taken together, individuals in honor cultures appear to be more likely to experience TBI, SCD, and heightened AD mortality, which might confer greater risk for ADRD. Additional research is required in this area to further assess ADRD risk within honor cultures, as individuals living within these cultures may benefit from culturally specific education and prevention efforts to reduce risk.

